# The Prevalence and Predictors of Low-Cost Generic Program Use in a Nationally Representative Uninsured Population

**DOI:** 10.3390/pharmacy4010014

**Published:** 2016-03-04

**Authors:** Joshua D. Brown, Nathan J. Pauly, Jeffery C. Talbert

**Affiliations:** Institute for Pharmaceutical Outcomes & Policy, Department of Pharmacy Practice and Science, University of Kentucky College of Pharmacy, 789 S. Limestone St., Lexington, KY 40506, USA; nathan.pauly@uky.edu (N.J.P.); jeff.talbert@uky.edu (J.C.T.)

**Keywords:** uninsured, generic drugs, low-cost generic, generic drug discount programs, MEPS, access

## Abstract

The uninsured population has much to gain from affordable sources of prescription medications. No prior studies have assessed the prevalence and predictors of low-cost generic drug programs (LCGP) use in the uninsured population in the United States. A cross-sectional study was conducted using data from the Medical Expenditure Panel Survey (MEPS) during 2007–2012 including individuals aged 18 and older who were uninsured for the entire 2-year period they were in MEPS. The proportions of LCGP fills and users was tracked each year and logistic regression was used to assess significant factors associated with LCGP use. A total of 8.3 million uninsured individuals were represented by the sample and 39.9% of these used an LCGP. Differences between users and non-users included higher age, gender, year of participation, and number of medications filled. The proportion of fills and users via LCGPs increased over the 2007–2012 study period. Healthcare providers, especially pharmacists, should make uninsured patients aware of this source of affordable medications.

## 1. Introduction

Individuals lacking medical insurance are disproportionately likely to suffer from negative health outcomes and barriers to health services than individuals with private or public insurance plans [1,2,3,4,5,6]. Prescription medications can offer a powerful first line of defense against conditions that commonly lead to costly in-patient hospital admissions, such as infections, heart disease and hypertension. However, despite their ability to ameliorate both chronic and acute illnesses, the high-cost of medications often serves as a barrier to access for uninsured individuals [7,8,9,10]. Low-cost generic drug programs (LCGPs) offered by major chain pharmacies improve both the affordability and accessibility of medications that may be used to treat many common acute and chronic conditions [11,12].

LCGPs first appeared in the United States (U.S.) in mid-2006 with Kmart providing 90 days of certain generics for $15 and was shortly followed by Wal-Mart’s $4 program [13]. Since then, generic discount programs are now in place at almost all major pharmacy chains including 8 of the top 10 largest chain pharmacies in the nation and include one-third of the top 100 generics used by Americans by volume [11,13,14]. These programs include a wide variety of medication categories—cardiovascular, antibiotics, asthma, analgesics, anti-diabetes, mental health, men’s health, and women’s health, among others [15,16,17].

Poor and uninsured individuals arguably have the most to gain from the increased affordability and accessibility offered by LCGPs. Despite the tremendous impact that LCGPs may have on the uninsured population in the U.S., there is a relative dearth of literature assessing the prevalence of LCGP use in a nationally representative uninsured population. In 2008, over 70 million Americans were estimated to have used an LCGP to obtain a prescription medication—a figure that has likely expanded as the number and popularity of these programs has increased [13,17,18]. In this 2008 self-reported survey, one-third of adults and one-quarter of children without insurance coverage reported using these programs [18]. However, given that self-reported surveys are particularly prone to recall and selection bias it is difficult to ascertain whether these estimates accurately represent LCGP use in the uninsured population.

Few studies have assessed the demographic and clinical characteristics of LCGP users and non-users [19,20] and none to our knowledge have assessed LCGP use specifically in an uninsured population. Understanding the factors that influence which individuals currently utilize these programs is a crucial first-step in increasing use amongst patients that have the most to gain from LCGPs. This study had four objectives: (1) Assess the prevalence of LCGP use in a nationally representative uninsured population; (2) Compare demographic and clinical characteristics of LCGP users and non-users; (3) Determine significant predictors of LCGP use; and (4) Analyze trends in LCGP use amongst uninsured individuals from 2007–2012.

## 2. Materials and Methods

### 2.1. Data Source

This study utilized public use data from the Medical Expenditure Panel Survey (MEPS) for the years 2007–2012. MEPS is a nationally representative survey of civilian, non-institutionalized individuals in the U.S. and includes information on demographics, healthcare utilization, medical conditions, and prescription medication use. MEPS uses an overlapping panel design with a new cohort (“panel”) added each year and participating in the survey for up to two years. Data are collected in five rounds throughout a panel’s two years of participation. Survey sampling and response weights are included so that population estimates may be obtained.

### 2.2. Study Subjects and Design

A cross-sectional study design was used to compare differences between LCGP users and non-users. Rates of LCGP use from 2007–2012 were quantified to assess trends in the proportions of LCGP fills and LCGP users over these years. These years were chosen because 2007 was the first full year in which LCGPs were available and 2012 is the most recent year of data available from MEPS. Inclusion in the study cohort required that individuals lack medical and pharmacy health insurance for both years of their MEPS panel, had no third party payers in the MEPS pharmacy component, participated in all five rounds of data collection, and reported using at least one prescription medication during their two-year panel period. The final sample was representative of those who are “long-term uninsured” (*i.e*., ≥2 years) who have filled a prescription.

### 2.3. Use of Low-Cost Generic Programs

MEPS captures medication fills at the pharmacy level; thus effectively capturing all medication fills paid by cash. Survey participants are first asked in-person about all medications which are have been filled. Surveyors obtain pharmacy information from prescription bottles and receive permission to contact the pharmacies for more information. Once permission is granted, details regarding each fill for each medication are extracted including name, NDC number, and the amounts paid by the customer or any third parties.

Four stipulations were used to define LCGP use: (1) The total cost of the drug was paid out of pocket; (2) The cost of the drug exactly matched the cost of an LCGP drug as reported by pharmacies; (3) The medication was listed on an LCGP formulary from a major chain pharmacy from 2007–2012; and (4) Oral medication fills were dispensed for 30 or 90 day supplies of medications, with the exception of anti-infectives, contraceptives, and steroids which were allowed to vary given differential dosing intervals for these classes. LCGP use was coded at the person level as a binary dependent variable for any use during the study period and at the medication level for each medication fill. Pharmaceutical utilization was determined for medications available from LCGPs based on Multum Lexicon classifications (Cerner Multum™, Denver, CO, USA), which is included in the MEPS pharmacy files for each medication.

### 2.4. Subject Characteristics

Cohort demographics and characteristics of interest included age, race, gender, family income level, number of prescriptions filled. For comparison, the cohort was divided by age categories: 0–17, 18–34, 35–54, 55–64, and 65+ years of age. Family income level was based on MEPS classifications of income levels which are stratified as a percentage of the Federal Poverty Level (FPL): <100%, 100%–125%, 126%–200%, 201%–400%, and >400% of the FPL. Residence within a metropolitan statistical area (MSA) was recorded as a binary variable and region was categorized by U.S. Census regions. Race was divided between Whites, Hispanics, African-Americans, Asians, and others. Age, family income, region, MSA and insurance type were all assessed at the last round of data collection. A Charlson Comorbidity Index (CCI) score was calculated for each individual based on the algorithm by Quan *et al.* [19].

### 2.5. Data Analysis

The proportions of LCGP uses and users were tracked from 2007 to 2012. These proportions were compared to overall pharmaceutical utilization for users and non-users over the same time period. Comparisons were conducted for cohort characteristics of interest between users and non-users using chi-square or *t*-tests. Multivariable logistic regression was used to identify factors associated with LCGP use. Adjusted odds ratios (AOR) and 95% confidence intervals were reported. All data analyses were conducted using SAS 9.4 [21] (SAS Institute, Cary, NC, USA) implementing SAS procedures (SURVEYMEANS, SURVEYFREQ, and SURVEYLOGISTIC) that take into account the complex survey design of MEPS and use the longitudinal survey weights supplied by MEPS to calculate population estimates over the two-year period. This manuscript was drafted in accordance with STROBE guidelines for reporting observational studies.

## 3. Results

### 3.1. Cohort Comparison

Over the years 2007–2012 a total of 2535 uninsured individuals met all inclusion criteria. Applying MEPS person weights, this population represents 8,327,690 uninsured individuals annually who filled a prescription medication—representing approximately one fourth of the U.S. uninsured population prior to the implementation of the Affordable Care Act. Of this population, 39.9% (*N* = 3,321,071) were classified as LCGP users, having filled at least one prescription through an LCGP during their two-year panel period.

Demographic and clinical comparisons between users and non-users are presented in Table 1. Significant differences between LCGP users and non-users existed in terms of age, sex, MEPS panel, number of medications filled, and number of unique medications used. LCGP users were significantly more likely to be aged 55 or greater than LCGP non-users (19.1% *vs.* 10.4%). Additionally, a significantly greater proportion of LCGP users were female than non-users (55.6% *vs.* 44.7%). In terms of the number of medications used, LCGP users filled significantly more unique prescriptions and had more total medication fills than non-users.

### 3.2. Predictors of LCGP Use

The full multivariable logistic regression model with adjusted odds ratios and 95% confidence intervals is presented in Table 2. Significant predictors of LCGP use included MEPS panel number and number of unique medications used. Participants in Panels 13–16—taking place from 2008–2012—had greater than double the odds of using an LCGP than individuals in panel 12 (2007–2008). The panel with the greatest odds of using LCGPs was observed to be panel 15 (2010–2011) with individuals in this panel being greater than four times more likely to use an LCGP than those in panel 12 (AOR: 4.02 (95% CI: 2.75–5.87)). Additionally, each unique medication used increased the odds of LCGP use by 43% (AOR: 1.43 (95% CI: 1.27–1.62)).

### 3.3. Medication Use

From 2007–2012 the study population filled a total of 20,143 prescriptions, with 81.7% of these prescriptions being available through LCGPs. Out of all medications available through LCGPs, only 34.7% were actually purchased through these programs. The majority of LCGP fills were for medications used to treat chronic conditions.

**Figure 1:**
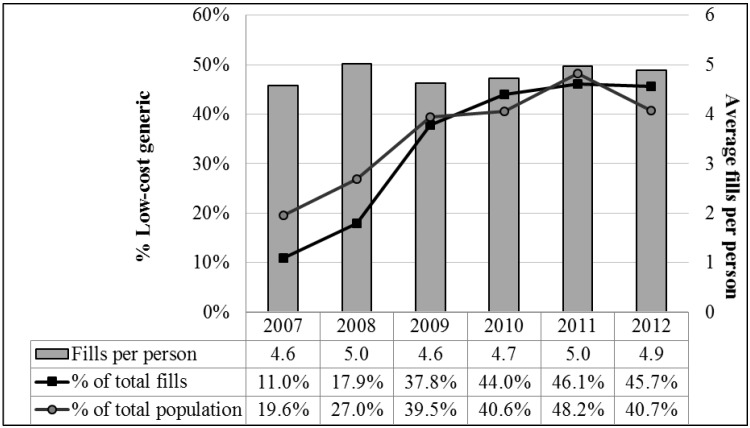
Proportion of LCGP fills, LCGP users, and total average prescription fills per person during the study period.

Trends in the use of LCGPs, including the proportions of LCGP fills and LCGP users in each year as well as the total number of prescription fills per person are presented in Figure 1. Proportions of both LCGP users and LCGP fills in each year increased significantly from 2007–2012. The proportion of prescriptions available through LCGPs that were obtained through LCGPs more than quadrupled from 11.0% in 2007 to 45.7% in 2012. Over the same time period, the proportion of LCGP users more than doubled from 19.6% in 2007 to 40.7% in 2012. While the proportions of LCGP users and LCGP fills both increased significantly from 2007–2012, overall pharmaceutical utilization remained relatively constant with approximately 4.8 prescription fills per person per year.

## 4. Discussion

Individuals living for long periods of time without insurance have the most to gain from the affordable medications offered by LCGPs—especially so for those also suffering from chronic illness or living below the poverty line. Results of this study indicate that the increasing use of LCGPs in the uninsured population is not due to increasing pharmaceutical utilization but rather due to increasing popularity and knowledge of these programs. However, despite the dramatic increase in LCGP use amongst the uninsured, it is disconcerting that fewer than 40% of uninsured individuals in the U.S. who use prescription medications are LCGP users. These numbers are comparable to insured adults and lower than elderly adults with Medicare [20]. Even when only considering individuals who filled at least one prescription available through LCGPs less than half actually filled that medication through an LCGP.

Uninsured individuals who are living below the federal poverty threshold are disproportionately likely to experience cost of prescription drugs as an impediment to medication access. One would hope that individuals living below the federal poverty level would use LCGPs at a greater rate than individuals better off financially. However, no significant differences were observed regarding the income distribution of LCGP users and non-users and income level was not a significant predictor of LCGP use. Taken together these results indicate that income and specifically poverty are not significant drivers of LCGP utilization.

The self-administered questionnaire in the MEPS household component includes questions regarding whether or not the participant experienced delays in getting necessary prescription medications, and if so, what the cause of the delay was. Given the widespread availability and relative affordability of LCGPs, one might expect that LCGP users would be less likely to experience delays in obtaining necessary medications. Our study found that there is not a significant difference between the number of LCGP users and non-users experiencing delays in obtaining prescription medications; nor do delays in obtaining medications make individuals significantly more or less likely to use LCGPs. Additionally, only 53.5% of individuals who reported cost of medication as the reason for a delay in obtaining medication were classified as LCGP users. Both of these results imply that LCGPs are not adequately being used to prevent delays in obtaining medication, particularly where cost is a prohibitive factor.

Many state and community public health service organizations supply prescription savings cards to uninsured individuals or include outreach programs to supply medications to the needy. These outreach services are a tacit reflection that public health entities understand the benefits of alleviating cost of medications as a barrier to medication access. In addition to these discount cards, public health entities should also actively promote the use of LCGPs and attempt to improve health literacy regarding the comparative efficacy of generic pharmaceutical products. Since many public health organizations already maintain online instructions regarding how to obtain prescription drug assistance, they can also include information about the widespread availability of LCGPs at little to no cost for the organization. Furthermore, prescribers as well as major chain pharmacies should take more of an active role in encouraging the use of LCGPs to treat common chronic and acute illnesses. These programs are being widely used by the rest of the population [20,22] and the implications of their use have been discussed elsewhere [11].

Given the recent expansion of Medicaid under the Affordable Care Act, many of the uninsured individuals included in this study will now be eligible for Medicaid benefits. Although these individuals will now receive prescription drug benefits through Medicaid plans, it is still vitally important to encourage LCGP use in this newly insured population. Previous research has demonstrated that individuals who are uninsured for long periods and subsequently gain insurance will dramatically increase their healthcare utilization and spending once they become insured [23,24,25]. Newly insured individuals under the Affordable Care Act should be encouraged to use LCGPs to decrease the barrier to effective pharmaceutical intervention where cost may still be a limitation even with insurance. This also has the potential to be cost saving for state and federal payers by offsetting the costs of prescription medications as well as decreasing clinical sequelae of conditions which the medications are meant to treat. Also, given the ongoing challenges to the Affordable Care Act, if it were to be totally dismantled, it would be very beneficial for the newly uninsured population to understand that affordable medications are available even without insurance coverage.

Our study is subject to some limitations. It may remain possible that not all medication use is recorded if all pharmacies used were not surveyed. Our study definition of LCGP use may allow for overestimation of use if only pricing is considered. However, this effect is mitigated by requiring specific quantities supplied for oral medications. Our algorithm passed face validity by not detecting medications that are not on LCGP formularies (e.g., benzodiazepines and narcotics) and also included estimates of generic use close to other estimates using surrogate markers of medication use (e.g., 10% of warfarin fills) [20,26]. We included a population representative of those who are uninsured for at least two continuous years and that have filled a prescription medication. Given the data source, we cannot verify the presence of unfilled medications in the population or account for those included in MEPS that did not report at least one medication fill. Finally, it is possible that some individuals exclusively use pharmacies in which LCGPs are not available and thus this population was never eligible for inclusion in the LCGP user cohort.

## 5. Conclusions

A significant proportion of individuals who were uninsured for two consecutive years from 2007–2012 used an LCGP during this period. Furthermore, the proportion of LCGP users and the proportions of LCGP fills out of all medications available through LCGPs both increased significantly from 2007–2012. Despite the tremendous gains in the rate of LCGP use, certain subgroups including individuals living below the poverty threshold and those experiencing delays in obtaining necessary medications could stand to benefit from greater use of these programs. State and community public health services should work alongside prescribers and major pharmacy chains to encourage greater use of LCGPs among the uninsured population.

## Figures and Tables

**Table 1:** Demographic characteristics of study cohort.

	LCGP Users				Non-Users			
	N	%	N	%	N	%	N	%
			(Weighted)	(Weighted)			(Weighted)	(Weighted)
*Overall Sample: N = 2535*								
	1023	40.4	3,321,071	39.9	1512	59.6	5,006,619	60.1
Age *								
0–17	55	5.4	197,217	5.9	130	8.6	468,222	9.4
18–34	308	30.1	1,097,996	33.1	597	39.5	2,060,812	41.2
35–54	465	45.5	1,461,488	44	637	42.1	1,955,761	39.1
55–64	180	17.6	531,079	16.0	144	9.5	509,644	10.2
65+	15	1.5	33,290	1.0	4	0.3	12,180	0.2
Sex *								
Male	413	40.4	1,473,855	44.4	753	49.8	2,766,344	55.3
Female	610	59.6	1,847,216	55.6	759	50.2	2,240,275	44.7
*Race*								
White	333	32.6	1,762,453	53.1	485	32.1	2,589,120	51.7
Hispanic	441	43.1	919,879	27.7	668	44.2	1,468,093	29.3
Black	187	18.3	429,532	12.9	252	16.7	659,446	13.2
Asian	38	3.7	118,957	3.6	59	3.9	157,624	3.1
Other	24	2.3	90,249	2.7	48	3.6	132,337	2.6
*Region*								
Northeast	70	6.8	270,591	8.1	153	10.1	568,708	11.4
Midwest	161	15.7	598,921	18.0	224	14.8	840,617	16.8
South	540	52.8	1,614,540	48.6	726	48	2,375,859	47.5
West	252	24.6	837,018	25.2	409	27.1	1,221,435	24.4
*Income Category*								
<100% of FPL	305	29.8	930,090	28.0	461	30.5	1,322,446	26.4
100% to 125% of FPL	86	8.4	230,157	6.9	151	10	400,395	8.0
125% to 200% of FPL	252	24.6	728,102	21.9	365	24.1	1,117,602	22.3
200% to 400% of FPL	274	26.8	900,861	27.1	387	25.6	1,396,285	27.9
>400% of FPL	106	10.4	531,861	16.0	148	9.8	769,891	15.4
*MSA*								
Rural	153	15	587,178	17.7	227	15	885,641	17.7
Urban	870	85	2,733,892	82.3	1,285	85	4,120,978	82.3
*Marital Status*								
Not Married	563	55	2,023,346	60.9	909	60.1	3,210,180	64.1
Married	460	45	1,297,725	39.1	603	39.9	1,796,439	35.9
*Employment*								
Unemployed	366	35.8	1,144,601	34.5	547	36.2	1,680,822	33.6
Employed	657	64.2	2,176,469	65.5	965	63.8	3,325,797	66.4
*Education*								
Less than High School	345	33.7	824,719	24.8	520	34.4	1,330,469	26.6
High School or Equivalent	481	47	1,721,585	51.8	668	44.2	2,402,929	48.0
Some College	197	19.3	774,767	23.3	324	21.4	1,273,222	25.4
MEPS Panel (years) *								
12 (2007–2008)	109	10.7	469,931	14.1	320	21.2	1,401,201	28
13 (2008–2009)	243	23.8	773,787	23.3	385	25.5	1,062,261	21.2
14 (2009–2010)	199	19.5	614,551	18.5	292	19.3	967,636	19.3
15 (2010–2011)	210	20.5	743,519	22.4	208	13.8	707,594	14.1
16 (2011–2012)	262	25.6	719,283	21.7	307	20.3	867,927	17.3
*Delays In Getting Necessary Medications*								
No Delays	919	89.8	2,918,531	87.9	1421	94	4,638,638	92.7
Delays	104	10.2	402,539	12.1	91	6	367,981	7.3
CCI *								
0–1	856	83.7	2,884,043	86.8	1425	94.2	4,736,873	94.6
2–4	160	15.6	424,906	12.8	65	4.3	214,697	4.3
5+	5	0.5	9106	0.3	4	0.3	9640	0.2
Missing	2	0.2	3016	0.1	18	1.2	45,409	0.9
Total Number of Medication Fills *								
Median (IQR)	5 (2–16)		4.9 (1.7–16.6)		2 (1–4)		1.3 (1–3.5)	
Unique Medications Used *								
Median (IQR)	3 (2–5)		2.1 (1.1–4.2)		1 (1–2)		1 (1–1.8)	

* *p* < 0.001 between group comparison; Percentages not adding to 100% are due to rounding errors; CCI = Charlson Comorbidity Index; IQR = interquartile range; MSA = metropolitan statistical area; LCGP = low cost generic program; FPL = federal poverty limit.

**Table 2:** Multivariable logistic regression results of predictive characteristics for LCGP use.

Characteristic	Adjusted Odds Ratio	95% Wald Confidence Limits	
*Age*		Lower	Upper
0–17	Ref.	Ref.	Ref.
18–34	1.12	0.63	2.00
35–54	1.20	0.65	2.22
55–64	1.50	0.75	2.97
65+	4.02	0.69	23.25
*Gender*			
Male	Ref.	Ref.	Ref.
Female	1.24	0.99	1.56
*Marital Status*			
Not Married	Ref.	Ref.	Ref.
Married	1.00	0.79	1.27
*Employment*			
Unemployed	Ref.	Ref.	Ref.
Employed	0.98	0.75	1.28
*Education*			
Less than High School	Ref.	Ref.	Ref.
High School or Equivalent	1.12	0.85	1.46
Some College	1.08	0.75	1.56
*Income Category*			
<100% of FPL	Ref.	Ref.	Ref.
100% to 125% of FPL	0.79	0.50	1.24
125% to 200% of FPL	0.96	0.73	1.28
200% to 400% of FPL	1.02	0.76	1.37
>400% of FPL	1.05	0.69	1.59
*Race*			
White	Ref.	Ref.	Ref.
Hispanic	1.18	0.89	1.56
Black	0.93	0.67	1.30
Asian	1.36	0.76	2.44
Other	1.18	0.63	2.19
*MSA*			
Rural	Ref.	Ref.	Ref.
Urban	1.11	0.80	1.53
*Region*			
Northeast	Ref.	Ref.	Ref.
Midwest	1.31	0.88	1.93
South	1.27	0.88	1.83
West	1.16	0.79	1.71
*Panel Number (years of panel)*			
12 (2007–2008)	Ref.	Ref.	Ref.
13 (2008–2009)	2.33	1.62	3.36
14 (2009–2010)	2.31	1.63	3.29
15 (2010–2011)	4.02	2.75	5.87
16 (2011–2012)	2.78	1.92	4.01
*Delays in Getting Necessary Medications*			
No Delays	Ref.	Ref.	Ref.
Delays	1.06	0.71	1.58
*CCI*			
0–1	Ref.	Ref.	Ref.
2–4	1.49	0.94	2.37
5+	0.72	0.16	3.18
*Number of Unique Meds*	1.43	1.27	1.62

CCI = Charlson Comorbidity Index; LCGP = low cost generic program; FPL = federal poverty level; MSA = metropolitan statistical area; Ref. = Reference category for variable.

## Abbreviations

The following abbreviations are used in this manuscript:

DOAJ	Directory of open access journals
FPL	Federal poverty limit
LCGP	Low-Cost generic program
LD	linear dichroism
MDPI	Multidisciplinary Digital Publishing Institute
MEPS	Medical Expenditure Panel Survey
MSA	Metropolitan Statistical Area
NDC	National Drug Code
TLA	Three letter acronym
U.S.	United States
